# Resistance to energy metabolism - targeted therapy of AML cells residual in the bone marrow microenvironment 

**DOI:** 10.20517/cdr.2022.133

**Published:** 2023-03-14

**Authors:** Yoko Tabe, Marina Konopleva

**Affiliations:** ^1^Department of Laboratory Medicine, Juntendo University, Tokyo 112-8421, Japan.; ^2^Department of Medicine (Oncology) and Molecular Pharmacology, Albert Einstein College of Medicine, Bronx, NY 10461, USA.; ^3^Department of Leukemia, The University of Texas MD Anderson Cancer Center, Houston, TX 77030, USA.

**Keywords:** Bone marrow microenvironment, acute myeloid leukemia, mitochondria, oxidative phosphorylation, fatty acid oxidation, energy metabolism

## Abstract

In response to the changing availability of nutrients and oxygen in the bone marrow microenvironment, acute myeloid leukemia (AML) cells continuously adjust their metabolic state. To meet the biochemical demands of their increased proliferation, AML cells strongly depend on mitochondrial oxidative phosphorylation (OXPHOS). Recent data indicate that a subset of AML cells remains quiescent and survives through metabolic activation of fatty acid oxidation (FAO), which causes uncoupling of mitochondrial OXPHOS and facilitates chemoresistance. For targeting these metabolic vulnerabilities of AML cells, inhibitors of OXPHOS and FAO have been developed and investigated for their therapeutic potential. Recent experimental and clinical evidence has revealed that drug-resistant AML cells and leukemic stem cells rewire metabolic pathways through interaction with BM stromal cells, enabling them to acquire resistance against OXPHOS and FAO inhibitors. These acquired resistance mechanisms compensate for the metabolic targeting by inhibitors. Several chemotherapy/targeted therapy regimens in combination with OXPHOS and FAO inhibitors are under development to target these compensatory pathways.

## INTRODUCTION

Acute myeloid leukemia (AML) comprises a highly aggressive, biologically heterogeneous group of hematopoietic disorders involving one or more cytogenetically or molecularly abnormal cell clones. It is primarily a disease of older adults. The standard of care for relapsed and refractory AML has progressed minimally in the past 30 years, with survival rates of less than 12% for patients over 65 years old^[1]^. Thus, novel therapeutic strategies that are more effective and carry a lower risk of organ damage than current treatments are urgently needed.

AML cells always face two major metabolic challenges: their high rate of proliferation imposes increased bioenergetic demands, and fluctuations in the availability of external nutrients and oxygen in the bone marrow (BM) microenvironment threaten cellular survival. In the BM, AML cells constantly modulate their metabolic state to adapt to this fluctuating microenvironment, shifting between quiescent, proliferative, and differentiated states^[2-4]^. Highly proliferative AML cells, drug-resistant AML cells, and leukemia stem cells (LSCs) that remain quiescent have been shown to depend on oxidative phosphorylation (OXPHOS). LSCs differ from bulk leukemia cells in that they possess stem cell characteristics including abnormal self-renewal capacity and drug resistance. Persistence of LSCs in the BM microenvironment after chemotherapy is considered an important factor in AML relapse^[5]^. Both quiescent AML cells and LSCs survive through metabolic activation of fatty acid oxidation (FAO) along with OXPHOS in their mitochondria. Hence, the reprogramming of energy metabolism processes in AML cells is recognized as a potential therapeutic target. Inhibition of OXPHOS and FAO can disrupt metabolic homeostasis, increase reactive oxygen species (ROS) production, and cause apoptosis in AML cells^[2,6,7]^. However, inhibition of this altered energy metabolism triggers various adaptive mechanisms in AML cells through their interaction with BM stromal cells. Thus, the BM microenvironment provides a setting in which secondary resistance to OXPHOS inhibition can develop, thereby contributing to the survival of AML cells. Therefore, strategies combining chemotherapy and specific molecular targeted therapy may have promise for eliminating BM-resident AML cells and LSCs.

In this review, we summarize the current state of knowledge about mitochondrial OXPHOS and fatty acid metabolism in residual AML cells in the BM microenvironment. We further describe the molecular mechanism by which AML cells acquire resistance to OXPHOS and FAO inhibitors. Finally, we evaluate potential therapeutic regimens combining OXPHOS and FAO inhibitors to target the metabolic vulnerabilities of BM-resident chemoresistant leukemia cells and LSCs.

## MAIN TEXT

### The BM microenvironment reprograms OXPHOS in AML cells

#### AML cells’ dependence on OXPHOS in the BM microenvironment

Whereas circulating AML cells are effectively eliminated by drug treatment, AML cells residing in the BM acquire resistance to chemotherapy. The BM microenvironment provides growth factors for leukemic cells, promotes immunosuppression, and supports leukemic cell survival. In response, leukemia cells adapt their metabolic state to this constantly changing environment^[7-9]^.

**Figure 1:**
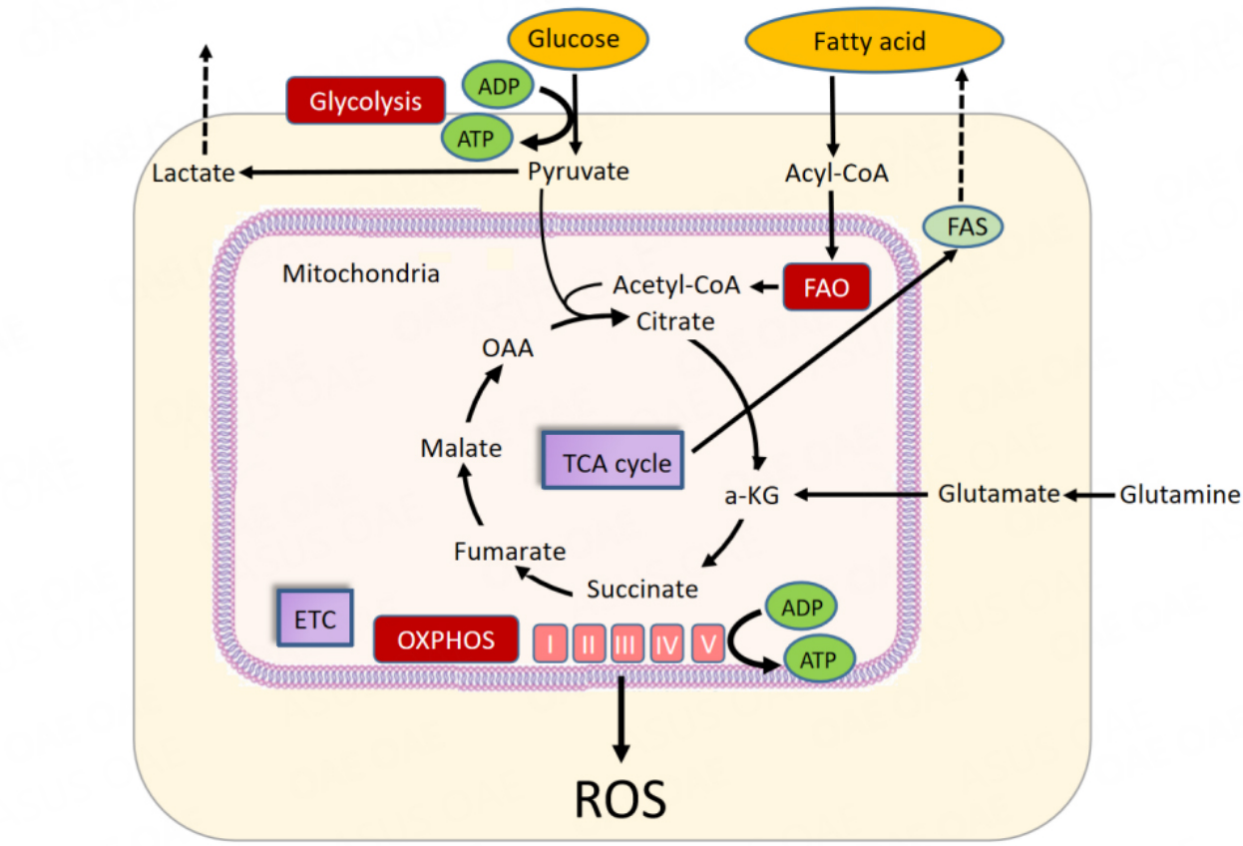
Energy metabolism in AML. Glucose is converted to pyruvate by glycolysis. Pyruvate is converted to acetyl-CoA for use in the tricarboxylic acid (TCA) cycle. ATP is produced by oxidative phosphorylation (OXPHOS) in the TCA cycle and electron transport chain (ETC). Fatty acid metabolism supplies acetyl-CoA to the TCA cycle via β-oxidation of fatty acids (FAO). Glutamine metabolism is the process of converting glutamine to glutamic acid. a-KG: Alpha-ketoglutarate; FAS: fatty acid synthase; OAA: oxaloacetic acid; ROS: reactive oxygen species.

Energy metabolism encompasses the molecular pathways whose products are involved in cellular energy production in the form of ATP. In leukemic cells, energy metabolism relies on OXPHOS and associated catabolic pathways, including glycolysis and fatty acid metabolism. The energy required for ATP production is produced by the mitochondrial potential, which causes protons to reenter the mitochondria through complex V. Fatty acid metabolism also supplies acetyl-CoA to the tricarboxylic acid (TCA) cycle through FAO [Figure 1].

Recently, the molecular mechanisms by which AML cells undergo metabolic reprogramming and those underlying the antileukemic efficacy of OXPHOS inhibitors have been demonstrated^[7,10]^. Actively proliferating AML cells respond to their increased energy and substrate demands via upregulation of OXPHOS, glycolysis, and related biochemical pathways. In turn, their bioenergetic efficacy strongly depends on extrinsic signals from the microenvironment^[11]^.

The leukemic BM microenvironment is generally hypoxic; during disease progression, hypoxic areas in the BM expand^[12-14]^. Indeed, AML cells so strongly depend on OXPHOS for metabolism that they might cause hypoxia in the BM environment. These hypoxic niches are expanded, in part, through activation of the transcription factor hypoxia-inducible factor 1α (HIF-1α)^[12,15]^. Transcriptional complexes often include metabolic enzymes, which locally supply substrates and cofactors to these complexes^[16]^. For this reason, OXPHOS itself and the transcription factors that regulate it are attractive targets for novel therapeutic interventions.

The persistence of LSCs and treatment-resistant AML cells in the BM remains the major cause of failure to eradicate AML. Cancer stem cells were initially identified in AML^[17,18]^ and subsequently validated in solid tumors. Across cancers, cancer stem cells share two important features: they can self-renew and produce differentiated progeny. The OXPHOS-dependent survival mechanism of LSCs is common to several solid tumor stem cells. For example, pancreatic cancer stem cells use OXPHOS for survival by accumulating the transcription coactivator peroxisome proliferator-activated receptor coactivator-1α (PGC-1α), which enhances mitochondrial biogenesis and the oxygen consumption rate and is sensitive to inhibitors of mitochondrial respiration^[19]^. Reliance on OXPHOS has also been observed in other solid-tumor stem cells, including those in brain^[10]^ and breast cancers^[20]^.

Several recent studies have revealed how AML LSCs exploit OXPHOS^[21]^. The transcriptional and epigenetic signatures of leukemia-initiating LSCs are largely mutation-independent^[22-25]^. Instead, rewired cellular metabolism has been increasingly recognized to play a significant role in LSC maintenance and treatment resistance in AML^[26]^. In LSCs, several components of the electron transport chain (ETC) complexes I and V have been shown to be more abundant than in normal hematopoietic stem cells (HSCs)^[27]^. Notably, AML LSCs overexpress antiapoptotic BCL-2, which has been shown to regulate ATP/ADP exchange across the mitochondrial membrane by facilitating regulation of voltage-dependent anion channels and adenine nucleotides,^[28,29]^ preventing the loss of coupled mitochondrial respiration during apoptosis^[30]^.

#### Rewiring of mitochondrial function facilitates AML resistance to OXPHOS inhibition

Understanding the crosstalk between AML cells and their microenvironment is critical to targeting the pathways involved in the metabolic reprogramming of chemoresistant AML cells and LSCs. In preclinical studies, most AML models responded to inhibition of OXPHOS via targeting of ETC complex I^[3]^. However, several clinical trials have shown that the efficacy of these OXPHOS inhibitors is limited^[31,32]^. In trials of several types of solid tumors, one putative complex I inhibitor, carboxyamidotriazole, had no clinical benefit^[31]^. Mouse studies of BAY87-2243, a novel complex I inhibitor, demonstrated antitumor activity and no toxic effects, but the phase I trial was terminated because of unexpected toxic effects^[32]^. These findings indicate that OXPHOS inhibitors have a narrow therapeutic window and emphasize the need to better understand how the BM microenvironment enables AML cells to become resistant to the metabolic stress caused by OXPHOS inhibition.

**Figure 2:**
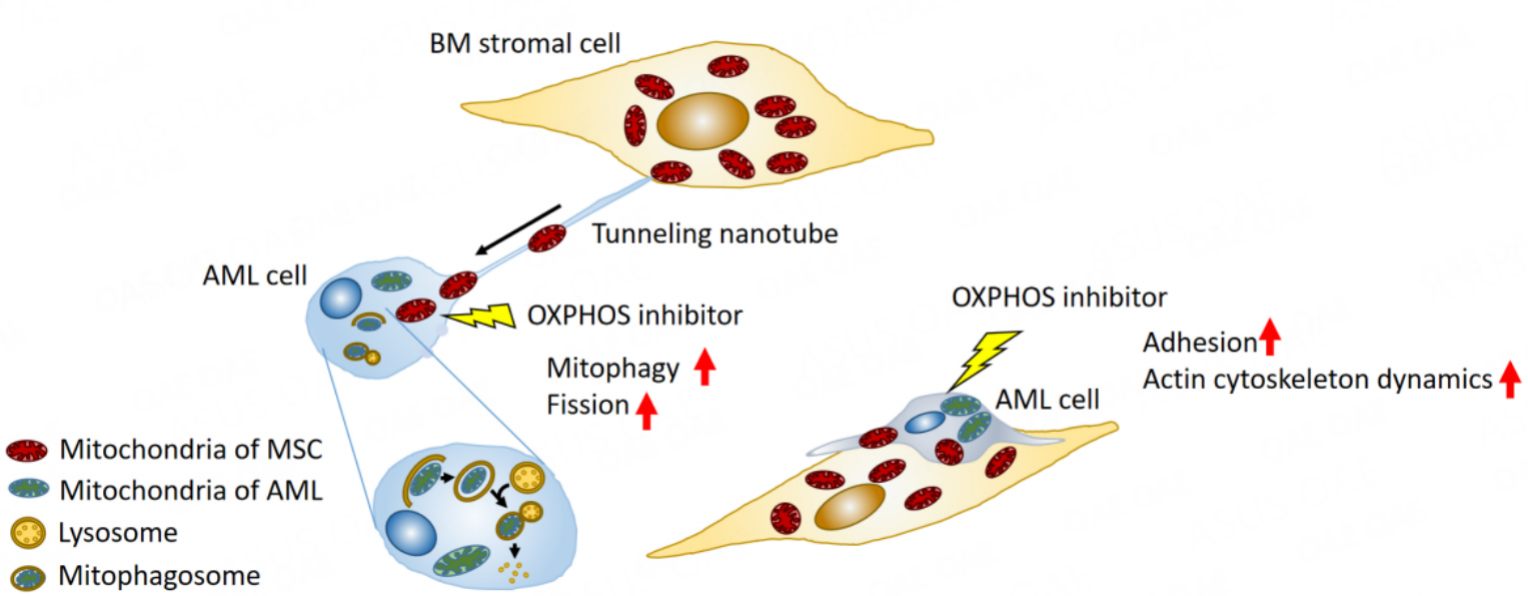
Secondary resistance to oxidative phosphorylation (OXPHOS) inhibition through bone marrow mesenchymal stem cells. OXPHOS inhibition stimulates cell adhesion and actin dynamics in AML cells. The bone marrow (BM) microenvironment facilitates the development of secondary resistance to OXPHOS inhibition by supporting direct mitochondrial trafficking via tunneling nanotubes from BM stromal cells to AML cells. This trafficking is accompanied by mitophagy and mitochondrial fission. MSC: Mesenchymal stem cell.

One such mechanism occurring in the tumor microenvironment is the horizontal transfer of mitochondrial DNA from host to tumor cells. Studies using *in vivo *models have shown that this transfer reestablishes respiration and promotes tumorigenesis^[33]^. In OXPHOS-dependent AML cells, OXPHOS inhibition induced formation of tunneling nanotubes that enabled this mitochondrial DNA trafficking from BM stroma cells to AML cells^[34]^. In the formation of tunneling nanotubes, a filopodium-like protrusion is extended from one cell to another^[35]^. This process is positively regulated by activation of motor proteins such as Rho GTPases through actin polymerization^[36,37]^ and filopodia formation through focal adhesion^[38^]. In addition, a recent study showed that a transmembrane complex gap junction channel opens under ROS-induced oxidative stress via PI3K-Akt activation to enable the transfer of mitochondrial DNA from stromal cells in the BM to HSCs [Figure 2]^[39]^.

OXPHOS inhibition-induced horizontal transfer of mitochondria from BM stromal cells to AML cells is accompanied by endogenous mitochondrial fission and elimination of damaged mitochondria by mitophagy, both of which contribute to AML cell survival^[34,40]^. As the process by which damaged mitochondria are segregated for elimination by autophagy, mitochondrial fission is central to mitophagy^[41-44]^. Cellular metabolism and cell survival require efficient mitophagy^[40,45]^. Because of the centrality of these processes in the maintenance of mitochondrial function, both mitochondrial fission and crosstalk with BM stroma cells via tunneling nanotubes may be another mechanism by which AML cells develop resistance to OXPHOS inhibition^[46]^.

LSCs have low rates of energy metabolism and cannot upregulate glycolysis after OXPHOS inhibition. Thus, they are particularly sensitive to OXPHOS blockade^[47,48]^. However, LSCs are able to maintain their stemness by mitochondrial fission and mitophagy, which balance mitochondrial functions such as energy production, ROS generation, and apoptosis regulation^[44]^. Given these competing pressures, LSCs have a fragile mitochondrial network. Thus, blocking both OXPHOS and other metabolic pathways is a promising strategy for overcoming OXPHOS resistance associated with the BM microenvironment. Two possible targets include the enzyme ASS1 and the lipid metabolism protein LRP1, both of which are overexpressed in OXPHOS inhibitor-treated AML cells *in vivo*. ASS1 is essential for the biosynthesis of arginine^[49]^, and LRP1 contributes to hemin-induced autophagy in leukemia cells^[50,51]^. Further investigations will improve our understanding of how these enzymes shape the responses of LSCs to the metabolic and energetic effects of OXPHOS inhibition.

Several repurposed drugs have been shown to inhibit OXPHOS. Biguanides, including metformin, are routinely used for diabetes treatment and have been proposed for use in cancer because they inhibit complex I of the ETC in cancer cells^[52]^. However, metformin carries a risk of severe lactic acidosis^[53,54]^, which is a safety concern for cancer patients. In addition, because high OXPHOS levels play a key role in cytarabine resistance, treatments combining cytarabine with OXPHOS inhibitors might be more effective than monotherapy with either type of agent^[33]^. Recently, a novel lipoate analog, devimistat, an inhibitor of two key TCA cycle enzymes, the pyruvate dehydrogenase and α-ketoglutarate dehydrogenase complexes^[55,56]^, showed a satisfactory safety profile in AML patients. In a phase I study, patients with relapsed or refractory AML who received a combination of devimistat with cytarabine and mitoxantrone had a complete remission rate of 50%^[57]^.

In a phase II study of this combination, responses were observed in older patients but not in younger patients. In addition, RNA sequencing analysis showed a decrease in expression of mitochondria-related genes with aging, suggesting that age-related reduction in mitochondrial quality may be related to devimistat response^[58]^. These are encouraging findings that indicate that this approach would be particularly effective for older patients with the highest unmet medical needs. In sum, the judicious use of novel OXPHOS inhibitors in combination treatments may add to armamentarium of currently available therapeutics.

### FAO metabolism of AML cells and LSCs in the fatty acid-abundant BM microenvironment

#### FAO of AML cells in the BM microenvironment

Adipocytes in the BM microenvironment support the survival of several types of tumor cells by stimulating FAO through fatty acid transfer^[59]^. While it was reported that BM adipocytes occupy approximately 60% of the BM in 65-year-old individuals^[60]^, the development of BM adipocytes varies across different skeletal regions, and single-point iliac biopsy may not represent the BM environment of the skeletal system containing the red marrow and yellow marrow. In a previous study, leukemic cells have been shown to colonize in both red and yellow marrow regions, adhere to the cortical bone in the spine, and have enhanced activity in the proximal/distal femur^[61]^. In addition, radiation therapy accelerates the differentiation of mesenchymal stem cells into adipocytes in BM^[62]^. Such temporal and spatial changes in the BM microenvironment may play a key role in leukemia’s dynamic adaptation of FAO and in leukemia cells’ interactions with BM stromal cells.

**Figure 3:**
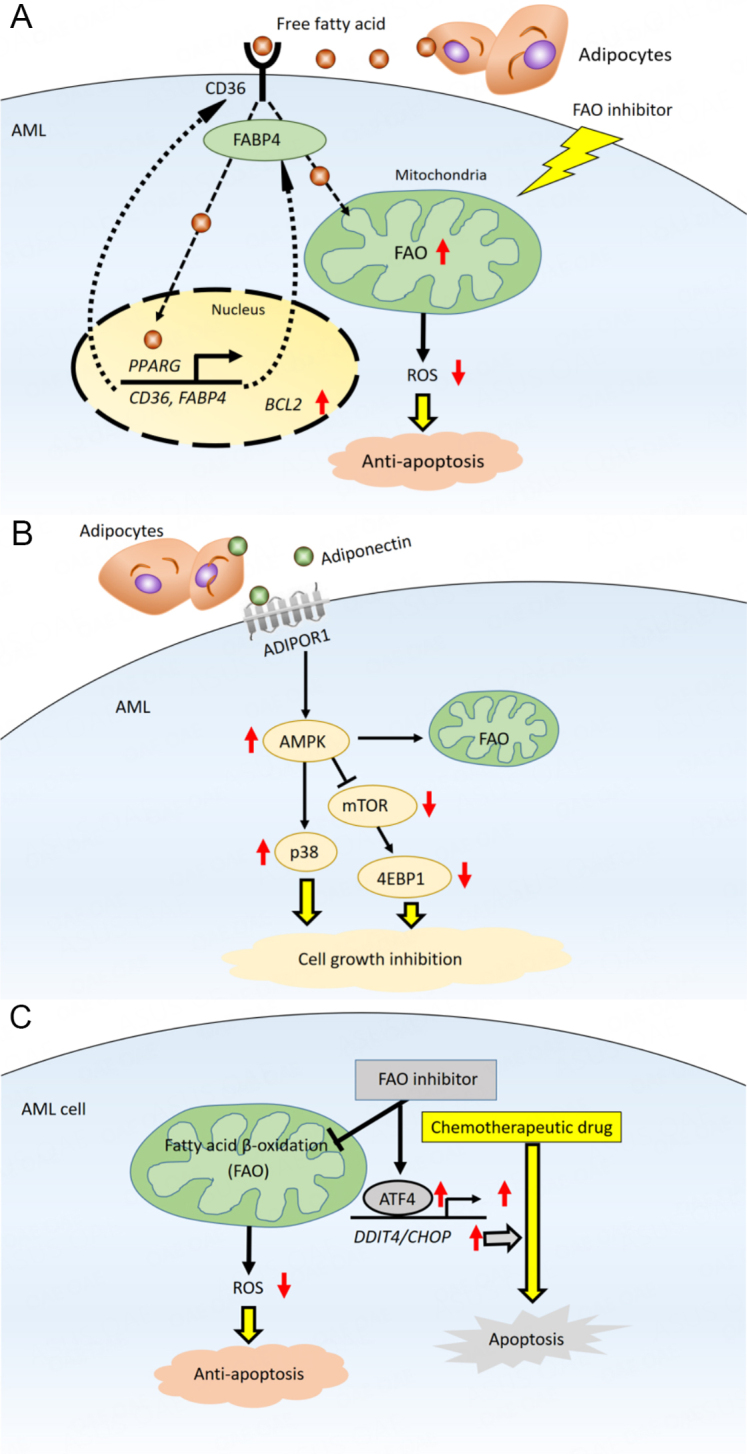
Bone marrow adipocytes promote fatty acid metabolism in AML. (A) Fatty acids derived through lipolysis of stored triglycerides in adipocytes induce upregulation of *PPARG*, *CD36*, and *FABP4* gene transcription, which stimulates fatty acid endocytosis. In mitochondria, fatty acids are metabolized through fatty acid oxidation (FAO), decreasing mitochondrial reactive oxygen species (ROS) formation and intracellular oxidative stress, thereby reducing apoptosis; (B) transcriptional regulation and fatty acid metabolism pathways maintain AML cells in a quiescent state. Activation of AMPK, upregulation of p38 and associated induction of autophagy, and upregulation of antiapoptotic HSP chaperone proteins in this state lead to chemoresistance; (C) in mitochondria, fatty acids are consumed for FAO, resulting in diminished formation of mitochondrial ROS and decreased intracellular oxidative stress. Inhibition of FAO induces an integrated stress response that stimulates transcriptional activation of ATF4 and promotes apoptosis induced by chemotherapy. ADIPOR1: Adiponectin receptor 1; ATF4: activating transcription factor 4; AMPK: AMP-activated protein kinase; FABP4: fatty acid binding protein 4; p38: p38 mitogen-activated protein kinase.

AML cells generally obtain fatty acids for FAO from the extracellular microenvironment through lipolysis of stored triglycerides^[63]^. FAO is metabolically activated to promote leukemic cell survival by remodeling and lipolysis of BM adipocytes. FAO is an essential source of mitochondrial NADH and FADH2 for the ETC and provides acetyl-CoA to the TCA cycle to produce ATP^[64]^. BM adipocytes supply long-chain fatty acids, which are then taken up into the cytoplasm via the scavenger receptor CD36^[65,66]^. Fatty acids activation is a two-step reaction. In the first step, the fatty acids form acyl-CoA in the cytoplasm. Then, FAO breaks down acyl-CoA to form acetyl-CoA in the mitochondria. Carnitine O-palmitoyltransferase 1 (CPT1) catalyzes a rate-limiting step of FAO; this enzyme conjugates fatty acids to carnitine, which is required for fatty acids to translocate from the cytoplasm to the mitochondria^[67]^. The internalized fatty acids are further transferred to the AML cell nucleus by the lipid chaperone fatty acid-binding protein 4 (FABP4). In the nucleus, the fatty acids ligate to peroxisome proliferator-activated receptor γ (PPAR)^[68]^. Activated PPAR induces downstream target genes, including *CD36*, *FABP4*, and the antiapoptotic *BCL2 *[Figure 3]^[69]^.

As with AML cells, the specific BM microenvironment created by the interaction between LSCs and stromal adipocytes supports the metabolic demands of LSCs. LSCs induce adipocyte lipolysis, which drives FAO in LSCs and facilitates their survival^[70,71]^. Therefore, CD36 and CPT1 are potential targets for AML. A CD36 neutralizing antibody inhibited metastasis of human melanoma and breast cancer cells^[72]^, and inhibition of CPT1 caused mitochondrial damage leading to cell death in primary AML cells^[67]^.

FABP4 is important in FAO and cancer cell survival in both solid and hematologic cancers. Adipocytes are known to serve as fatty acid reservoirs in breast cancer and melanoma^[73,74]^. Ovarian cancer cells also survive and proliferate in an adipocyte-rich microenvironment^[75]^. When primary human omental adipocytes were co-cultured with ovarian cancer cells, the adipocytes underwent lipolysis, and FAO was induced in the cancer cells^[63]^. These processes are mediated by adipokines including interleukin-8 and by upregulation of FABP4 both in adipocytes and ovarian tumor cells. In studies of leukemia, AML cells co-cultured with BM adipocytes exhibited higher levels of FABP4^[69]^, and knockdown of FABP4 prolonged survival in a mouse model of leukemia^[71]^. Thus, FABP4’s critical role in cancer cell survival involves its interactions with adipocytes.

Activation of β-adrenergic receptors, along with a G protein-coupled cascade that stimulates the lipolytic enzyme hormone-sensitive lipase (HSL), induces lipolysis of adipocytes^[76,77]^. Ovarian cancer cells have been found to upregulate HSL phosphorylation, thereby stimulating the release of free fatty acids from adipocytes^[63]^. AML blasts also induce HSL phosphorylation and, thus, activation of lipolysis in BM adipocytes^[71]^.

BM adipocytes also increase AML cells’ expression of adiponectin and its downstream target, AMP-activated protein kinase (AMPK), a stress response kinase^[69]^. AMPK, an important modulator of energy metabolism, is activated upon ATP depletion. Its functions include upregulation of fatty acid uptake, FAO, and regulation of autophagy^[78,79]^. Levels of adiponectin, much of which is supplied by BM adipocytes, have been shown to increase during cancer therapy^[80]^, and to promote chemotherapy resistance in myeloma cells via inducing adipokine secretion of adipokines and activating AMPK-dependent autophagy^[81,82]^. AMPK also positively regulates responses of an antiapoptotic chaperone heat shock protein that binds to denatured and unfolded proteins and promotes protein refolding or degradation to support AML cell survival^[79]^. In sum, leukemic cells often rely on fatty acids when they undergo metabolic stress, and the NADH and FADH2 generated by FAO support ATP production, redox homeostasis, biosynthesis, and cell survival.

FAO is involved in the interactions between LSCs and BM stromal cells^[83]^. LSCs rely on fatty acid uptake and consumption to shape their adaptation to the conditions of the BM microenvironment, their response to drugs, and their development of drug resistance^[69]^. Several such mechanisms have been identified. Mitochondrial uncoupling in AML cells negatively regulates Bak-dependent mitochondrial permeability transition^[84]^. In a study using samples from patients with relapsed AML, LSCs acquired the ability to counteract the loss of amino acid metabolism by upregulating FAO^[85]^. Specifically, this mechanism may underlie the development of resistance to treatment with azacitidine/venetoclax, a common induction regimen used mainly in older patients with AML^[85,86]^. In addition, a preclinical study demonstrated that cytarabine-resistant AML cells had enhanced FAO and OXPHOS^[66]^. Thus, targeting the metabolic vulnerabilities of chemoresistant LSCs, such as their dependence on FAO, may be a useful strategy for eradicating these cells.

#### FAO inhibitors and resistance acquired by compensatory metabolism in the BM microenvironment

FAO inhibition disrupts metabolic homeostasis, increases ROS levels, and induces expression of the integrated stress response mediator ATF4 in AML cells, all of which contribute to apoptosis^[87]^. Numerous studies have reported the anti-AML effect of inhibition of CPT1, the major rate-limiting enzyme in FAO^[67,84,87,88]^. CPT1 positively controls FAO by conjugating fatty acids with carnitine to transfer fatty acids into the mitochondrial matrix. Etomoxir is a pharmacological inhibitor of CPT1A, one of the isoforms of CPT1^[89]^, frequently used to block free fatty acids from entering the mitochondria via CPT1. Although the clinical use of etomoxir has ceased because of adverse effects^[90]^, the CPT1 inhibitor perhexiline can sensitize breast cancer cells to paclitaxel^[91]^, and other CPT1 inhibitors^[92]^ are currently being investigated for use in cancer therapy. The CPT1A inhibitor ST1326 has been shown to cause cell growth arrest, mitochondrial damage, and apoptosis in AML cells in a dose- and time-dependent manner^[67]^. Another novel FAO inhibitor, avocatin B, which is derived from avocados, decreased NADPH levels that were increased by FAO through acetyl-CoA and NADH production, inducing ROS-dependent cell death in AML cells^[93,94]^. Finally, the fatty acid synthase inhibitor orlistat has induced apoptosis in leukemic cells^[2]^.

The intramitochondrial FAO enzyme very long-chain acyl-CoA dehydrogenase (VLCAD) is critical in supporting both FAO and OXPHOS in AML cells and LSCs. Recently, preclinical studies have demonstrated the antileukemia activity of a novel small-molecule VLCAD inhibitor, a polyhydroxylated fatty alcohol with a terminal alkyne (AYNE)^[95]^. AYNE reduced mitochondrial respiration by altering FAO, which led to reduced ATP production in AML cells, even though AYNE also moderately upregulated glycolysis. In a mouse model, pharmacological inhibition of VLCAD with AYNE significantly reduced the repopulation potential of leukemia cells and was well tolerated^[95]^. Notably, normal HSCs compensate for this reduced replicative capacity through glycolysis which maintains their ATP levels and thus their viability^[95,96]^. These findings demonstrate the importance of focusing on the specific metabolic vulnerabilities of residual AML cells and LSCs that survive chemotherapy-induced stress. Unfortunately, only a few FAO inhibitors have advanced from preclinical to clinical studies [Table 1].

**Table 1:** FAO inhibitors in clinical trials on cancer treatment

**Compounds**	**Targeted enzyme**	**Clinical applications**	**Phase**	**ClinicalTrials.gov Identifier**	**Verified**
Trimetazidine	3-ketoacyl-C3-ketoacyl-CoA thiolase	Advanced Hepatocellular Carcinoma	Phase 3	NCT03278444	September 2017
Trimetazidine	3-ketoacyl-C3-ketoacyl-CoA thiolase	Intermediate-stage Hepatocellular Carcinoma	Phase 3	NCT03274427	September 2017
Ranolazine	3-ketoacyl-C3-ketoacyl-CoA thiolase	Prostate Cancers	N//A (Pilot Study)	NCT01992016	December 2018

Because AML is heterogeneous and multiclonal, blocking only one part of this complex metabolic system may allow residual cells to adapt metabolically. For instance, it has been shown that BM-derived stromal cells, including adipocytes, diminish the antileukemia effects of FAO inhibitors in AML cells by increasing glycolysis and glucose and free fatty acid uptake^[87]^. This compensatory induction of glycolysis is a sustained source of ATP to AML cells and, in turn, induces substantial lactate production. Similarly, FAO-deficient *Abcb11*-knockout mice exhibited high FABP4 and CD36 expression and free fatty acid uptake^[97]^. In sum, it is clear that FAO inhibition initiates several different adaptive mechanisms that promote AML cell survival in the BM microenvironment.

For this reason, treatment options based on combination regimens have been tested. Although FAO inhibition alone can trigger compensatory activation of other metabolic pathways, FAO inhibitors can also synergize with conventional antitumor agents such as paclitaxel^[91]^. For example, FAO and OXPHOS are increased in cytarabine-resistant AML cells; FAO inhibition with etomoxir induced a metabolic shift from high to low OXPHOS, sensitizing the cells to cytarabine^[66]^. Similarly, the combination of avocatin B and cytarabine synergized to enhance ROS production and induce apoptosis in AML cells co-cultured with BM adipocytes^[87]^. The role of avocatin B in apoptosis induction was attributed to activation of endoplasmic reticulum stress-induced ATF4^[87]^. These findings suggest that AML cells treated with cytarabine exhibit increased dependence on FAO, which may account for the synergism of cytarabine and FAO inhibitors.

## CONCLUSIONS

AML cells and LSCs both strongly depend on the production of mitochondrial biomass and on OXPHOS^[66]^ and FAO^[84]^ for survival. Compared to healthy HSCs, AML cells and LSCs are more susceptible to mitochondrial stress because their respiratory chain reserve capacity is lower^[98]^. These characteristic differences in the metabolism of AML cells and their normal hematopoietic-cell counterparts represent a specific vulnerability of leukemia cells and therefore are drawing a great deal of attention as targets for AML therapy. The results of studies in preclinical models using agents that target fatty acid metabolism have been encouraging.

Although they are vulnerable to the targeting of their metabolic pathways, AML cells, drawing on microenvironmental factors, can adapt to metabolic stress by activating metabolic bypass processes. Several *in vivo* studies and clinical trials have shown that the use of metabolic inhibitors alone is ineffective both because of their narrow therapeutic window and because of these adaptive mechanisms^[87]^. Alternatively, inhibition of FAO and other metabolic mechanisms along with conventional chemotherapy or targeted therapy may synergistically eradicate chemotherapy-resistant AML cells present in the BM.

In conclusion, understanding AML cell metabolism in the specific context of the BM microenvironment is crucial to improving therapies for AML. Because characteristics of the BM microenvironment enable the acquisition of resistance to OXPHOS and FAO inhibitors, drug combination strategies that interfere with these adaptations are needed. A more comprehensive understanding of the mechanisms of AML cell metabolism in future studies may reveal new treatment options targeting OXPHOS and FAO, enhance the efficacy of chemotherapeutic agents that target related pathways, reduce the toxicity of these agents, and enable the translation of new combinations of agents into clinically applicable treatment strategies.

## DECLARATIONS

### Copyright

© The Author(s) 2023.
